# Investigation of association between hip morphology and prevalence of osteoarthritis

**DOI:** 10.1038/srep23477

**Published:** 2016-03-22

**Authors:** Wei-Nan Zeng, Fu-You Wang, Cheng Chen, Ying Zhang, Xiao-Yuan Gong, Kai Zhou, Zhi Chen, Duan Wang, Zong-Ke Zhou, Liu Yang

**Affiliations:** 1 Center for Joint Surgery, Southwest Hospital, Third Military Medical University, Chongqing, 400038, China; 2 Department of Orthopaedics, West China Hospital, Sichuan University, Chengdu, 610041, China

## Abstract

The cause of hip osteoarthritis (OA) remains unclear, morphologic abnormality of hip was thought to be a contributing factor to hip OA. The hypothesis was that there were subtle anatomical morphology differences of the hip between normal and OA subjects; the objective of this study was to explore these anatomical differences which are predisposing to hip OA based on CT 3D reconstruction. Ninety-three normal subjects (186 hips) and 66 mild-to-moderate hip OA subjects (132 hips) were recruited in this study. Three parameters of the head-neck relationship were assessed: translation, rotation and concavity. Translation was the potential translational movements of femoral head related to the neck’s axis. Rotation was described by the physeal scar to evaluate the rotation tendency of femoral head related to the neck at the head-neck junction. Concavity was used to assess the sphericity of the head as it joins the neck. The femoral neck anteversion angle and some parameters of the acetabulum: anteversion, inclination and CE angle were measured too. By comparison, it was found that OA subjects had less femoral head sphericity, head-neck junction concavity, acetabular and femoral neck anteversion angle; but greater acetabular coverage. These characteristics increased the risk of hip OA in OA subjects.

Osteoarthritis (OA) is a chronic degenerative joint disorder with high prevalence. The cause of this disease remains unclear; several risk factors for hip OA have been identified such as ageing, obesity, overuse, male sex, joint trauma and so on^1^,^2^,^3^. OA usually affects individuals aged 55 years and above, leading to joint pain, stiffness, and physical disability^4^. Currently, there is no effective cure for OA and treatments are mainly focused on relieving pain and improving function^5^,^6^. A better understanding of the disease may help develop more effective treatments in the future.

Studies have shown that congenital or developmental anatomical abnormalities such as developmental dysplasia of the hip (DDH), Perthes disease, and slipped capital femoral epiphysis (SCFE) may cause hip OA in young adult patients^7^. Mild hip dysplasia and femoroacetabular impingement (FAI) are important causes of hip OA in elderly adult patients^8^,^9^. Hip dysplasia may result in abnormal mechanics distribution and instability in hip joint, and femoroacetabular impingement may lead to microtrauma of hip^10^,^11^. In addition, a close correlation was reported between OA and femoral head-tilt, pelvic incidence (PI), acetabular overcoverage, femoral head asphericity or reduced acetabular and femoral anteversion^12^,^13^,^14^,^15^. It was found that pelvic incidence correlated with acetabular retroversion as well^16^. Furthermore, the prevalence of OA has racial and geographic distribution differences^17^,^18^. Hip OA prevalence in Caucasian groups is approximately ten times the prevalence of hip OA in Chinese populations of the same age and gender^19^. Dudda reported that Caucasians might be at higher risk of hip OA than Chinese because of morphological findings that predisposed them to FAI^20^. Therefore, anatomical factors of the hip have an important role in the pathogenesis of hip OA.

Although the anatomical feature of the hip joint has been scrutinized by numerous previous studies, these studies were mainly based on supine anterior-posterior (AP) pelvis radiographs, and few studies has focused on the relationship between anatomical morphology and pathogenesis of hip OA. In order to further explore this concern, we conducted this study which was based on 3D CT parameters of the hip joint of normal and OA samples from Chinese people.

## Materials and Methods

### Subjects and scanning

We collected ninety-three normal patients (186 hips) from 20 to 50 years old, who had a CT scan due to reasons other than hip joint diseases between July 2011 and May 2014. The exclusion criteria was: evidence of any abnormalities of the joint including degenerative changes, a history of hip trauma or infection, rheumatic diseases, aseptic necrosis of the femoral head, and previous hip surgeries. A control group of sixty-six (132 hips) mild-to-moderate bilateral OA patients 20 to 50 years old were collected. Diagnostic criteria were according to hip OA diagnostic criteria of American College of Rheumatology. Degree of OA was determined using the Tonnis classification of OA^13^. The exclusion criteria were: OA of the hip that secondary to conditions such as osteonecrosis, trauma, sepsis, rheumatoid arthritis, hip dysplasia and slipped capital femoral epiphysis. There was no statistical difference with respect to sex, age, weight, height and BMI between these two groups. (Table 1) The study was approved by the ethics committee of Sichuan University. All the methods involving human subjects were carried out in accordance with relevant approved guidelines and regulations. Informed consent was obtained from each patient.

Scanning was carried out on a 64-layer spiral CT scanner (Somatom Sensation 64; Siemens Medical Solutions, Erlangen, Germany) from the iliac crest to the knee. Patients lay on the CT bed in a supine position with lower extremities in a neutral (with feet fixed at 15–30 degrees of internal rotation), horizontal position and parallel to each other. Scanning parameters were: 120 kV; 200–250 mA; layer thickness 1.0 mm; and reconstruction interval 0.75 mm. The data were transmitted to a Siemens syngo images post-processing work station to obtain 3D reconstructed images which could be measured in any plane and any direction. Measurements were carried out by two independent researchers.

### Study of hip morphology

**Figure 1:**
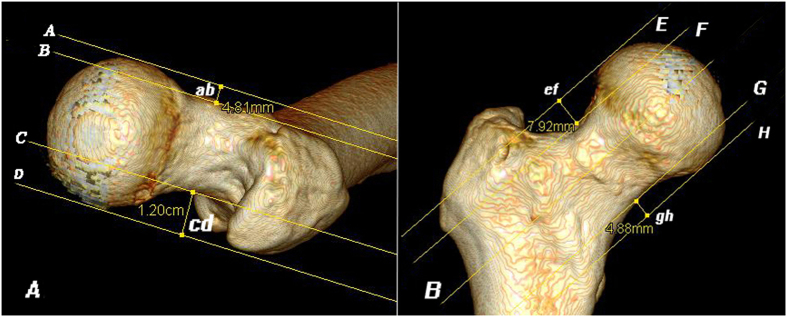
Head-neck translation measurements. The AOS was defined as the perpendicular distance (ab) between Lines A and B, Line A was drawn parallel to the neck axis and tangential to the convexity of the femoral head; Line B was drawn parallel to the neck axis and tangential to the concavity of the femoral neck^21^. Similarly, the POS was defined as the perpendicular distance (cd) between Lines C and D, SOS was defined as the perpendicular distance (ef) between Lines E and F, and IOS was defined as the perpendicular distance (gh) between Lines G and H. (AOS, anterior offset; POS, posterior offset; SOS, superior offset; IOS, inferior offset).

The first step was to define the anterior-posterior (AP) and lateral plane. In the AP plane, the femur was put in such a position that the axis of femoral neck was parallel to the coronal plane by rotating the femoral shaft internally with the axis of femoral shaft vertical to the horizontal plane. In the lateral plane, the femur was put in such a position that the axis of femoral neck was parallel to the coronal plane with the convex surfaces of the medial, lateral condyle and the posterior apex of greater trochanter in the same horizontal plane (Fig. 1). The main parameters we measured were three parameters of the head-neck relationship (translation, rotation, concavity) and the neck-shaft relationship (neck anteversion), as described by Toogood^21^. Other measured parameters included acetabular anteversion, inclination, and central-edge (CE) angle.

The parameter of the head-neck translation was used to indicate shifts or translations of the femoral head relative to the axis of the neck in AP and/or superior-inferior vectors, because the center of the femoral head normally is not aligned on the axis of the femoral neck. We used four offset measurements based on descriptions by Ito^22^, Siebenrock^23^ and Toogood^21^ on AP and lateral planes, including: anterior offset (AOS); posterior offset (POS); superior offset (SOS); and inferior offset (IOS) (Fig. 1). Then we used offset ratios AOS/POS and SOS/IOS to evaluate the translations of the head relative to the axis of the neck in AP and/or superior-inferior vectors. For example, if the AOS/POS and/or SOS/IOS offset ratio was equal to 1, it would means that the femoral head had no translation in the anterior-posterior and/or superior-inferior vector; if femur with AOS/POS and/or SOS/IOS ratio less than 1, it would mean that femoral head was translated posteriorly and/or inferiorly. If the AOS/POS and/or SOS/IOS offset ratio was greater than 1, the femoral head would be translated anteriorly and/or superiorly.

**Figure 2:**
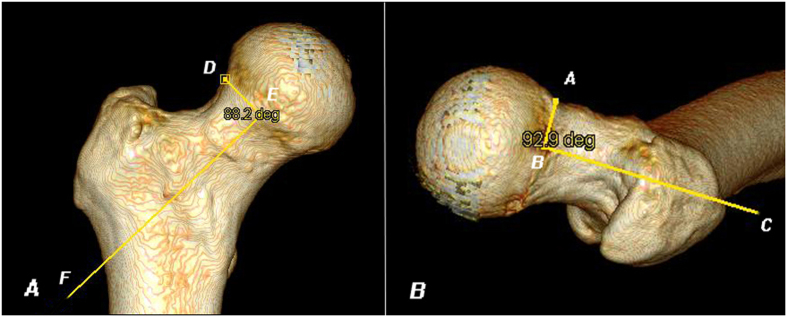
Head-neck rotation measurements. The AP physeal angle was defined as the acute, superior-lateral angle between Lines DE and EF. Line DE represented the physis; Line EF represented the neck axis^21^. Similarly, the lateral physeal angle was defined as the acute, superior-lateral angle between Lines AB and BC. Line AB represented the physis; Line BC represented the neck axis.

The parameter of head-neck rotation was used to indicate rotational movements of the head relative to the neck axis. As reported by Toogood^21^ the physeal scar was not perpendicular to the axis of the femoral neck, and the femoral head was abducted in the AP plane and anteverted in lateral plane relative to the femoral neck in Caucasians. In order to quantify these rotational movements in Chinese people, we used the physeal angles described by Toogood (Fig. 2). Physeal angle of 90˚ would indicate that the femoral head had no rotation relative to the neck axis.

**Figure 3:**
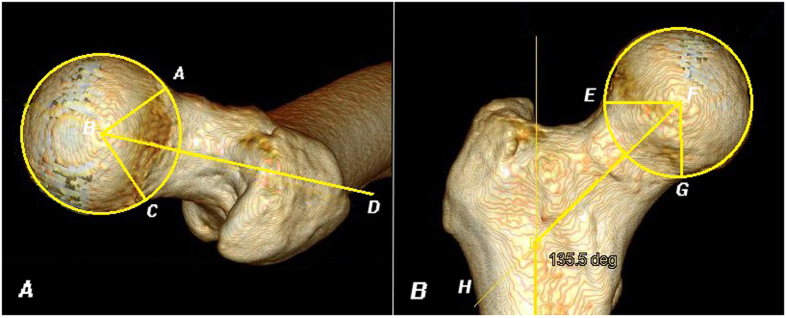
Head-neck junction concavity measurements. The alpha angle was defined as the acute angle between Lines AB and BD. Line AB was formed by connecting the center of the femoral head (Point B) and the point where the cortical surface of the head-neck junction first exited a perfect circle drawn around an ideally spherical femoral head (Point A)^21^; Line BD represented the neck axis. Similarly, beta angle was defined as the acute angle between Lines BC and BD, gamma angle was defined as the acute angle between Lines EF and FH and delta angle was defined as the acute angle between Lines FG and FH.

The third parameter of the head-neck relationship examined was the head-neck junction concavity. Because the femoral head is aspheric, not a perfect sphere as we thought, the femoral neck is not a perfect cylinder also. The sphericity of the head at the head-neck junction is irregular; a head-neck junction that is too broad or aspherical may cause cam-type impingement^22^. Concavity was used to assess the sphericity of the head as it joins the neck. Four angles were used: alpha and beta angle in the lateral plane; gamma and delta angle in the AP plane (Fig. 3). Smaller angle represented greater concavity at the head-neck junction and therefore nearly spherical femoral head, and lower probability of cam-type FAI. On the contrary, larger angle indicated less concavity, and a higher probability of cam-type FAI.

**Figure 4:**
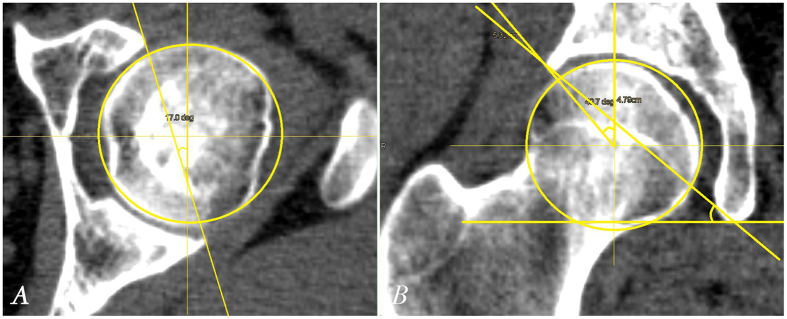
Acetabular anteversion, acetabular inclination, and the CE angle measurements. (**A**) Acetabular anteversion was measured on the axial view including the femoral head center. (**B**) Acetabular inclination and CE angle were measured on the coronal view including the femoral head center.

Besides, to achieve long-lasting good function of the hip joint, optimum femoral head coverage by the acetabulum is required^11^. Lack of coverage can lead to instability and overloading, while over-coverage such as acetabular retroversion and acetabular protrusion may lead to pincer-type FAI. Therefore, we measured acetabular anteversion, inclination, and CE angle, using previously described methods^24^,^25^ (Fig. 4).

### Statistical analysis

The data was expressed as mean ± standard deviation (SD). Student’s t-test and chi-squared test were used to analyze the two groups where appropriate with *p* < 0.05 being considered significant. The statistical analysis was performed using SPSS 13.0 (SPSS Inc, Chicago, USA). Thirty cases were randomly selected for the assessment of inter-observer and intra-observer agreements. Two observers independently assessed each anatomical parameter from the 30 cases to assess inter-observer agreement. The first observer also re-measured each anatomical parameter from the same 30 cases 4 weeks later to allow an assess intra-observer agreement. The intra- and inter-observer agreements were assessed according to the Bland and Altman method^26^,^27^. This method calculates 95% limits of agreement (LOA). The statistical analysis was performed by MedCalc statistical package (MedCalc Software, Mariakerke, Belgium).

## Results

The parameters of head-neck translation and rotation were displayed in Table 2. On the whole, in normal group, average AOS/POS and SOS/IOS ratio was greater than 1. Thus, the femoral heads of the population were, on average, tended to be translated anteriorly and superiorly. In the OA group, the average value of AOS/POS and SOS/IOS ratio were smaller than that of the normal group, specifically the average SOS/IOS ratio of the OA group was less than 1. Furthermore, female subjects tended to have a greater AOS/POS and SOS/IOS ratio in comparison with the male subjects, except for the AOS/POS ratio in OA group (*p* = 0.759). As for AP and lateral physeal angle, the mean value, was less than 90°. Femoral heads of the population were rotated with respect to the neck axis, on average, abducted and anteverted. Female subjects had a smaller AP and lateral physeal angle in comparison with the male subjects, but this gender difference had no significant difference except for the AP physeal angle in the normal group. In the OA group, subjects had greater AP and lateral physeal angles, indicating the femoral heads of the population were tended to be rotated more neutral.

The parameters of head-neck junction concavity were displayed in Table 3. On the whole, the normal group had a smaller alpha, beta, gamma and delta angle in comparison with the OA group, the normal group femoral head-neck junctions had, on average, greater concavity anteriorly, posteriorly, superiorly and inferiorly. Similarly, female subjects had a smaller alpha, gamma and delta angle in comparison with the male subjects in both groups, indicating female subjects had a greater head-neck junction concavity anteriorly, superiorly and inferiorly.

The parameters of acetabulum and femoral neck anteversion angle were shown in Table 4. It displayed that normal subjects had bigger acetabular and femoral neck anteversion angles, but smaller acetabular inclination and CE angles in comparison with the OA subjects. Meanwhile, in comparison with the male subjects, female subjects had bigger acetabular and femoral neck anteversion angles, but smaller acetabular inclination and CE angles, while these differences were not significant in acetabular anteversion and inclination angles in OA group (*p* = 0.429 and *p* = 0.080).

The Bland and Altman method showed a good intra- and inter-observer agreement on the anatomical parameter measurements. Differences and LOAs for each anatomical measurement for intra-observer and inter-observer reproducibilities were summarized in Table 5.

## Discussion

Some previous studies have tried to clarify the pathomechanism of hip OA from the perspective of anatomy, but most of the evaluations were mainly based on the examination of AP plain films, which may not be accurate enough^20^. In this study, we used 3D reconstruction technology to assess the relationship between femoral head and neck in terms of translation, rotation, and concavity, as well as femoral neck anteversion and some parameters of acetabulum. Then, we compared the major differences between normal and OA subjects, male and female subjects.

In this study, the average ratio of AOS/POS and SOS/IOS were greater than 1 (except for the average SOS/IOS ratio in OA group). The average ratio in OA subjects was less than that of the normal subjects which means femoral heads were translated more posteriorly and inferiorly in OA subjects. As for the rotation of femoral heads, OA subjects had bigger AP and lateral physeal angles. Therefore, femoral heads of the OA subjects were, on average, rotated more adducted and retroverted with respect to the neck axis when compared with normal subjects. These translation and rotation tendency would, in theory, increase anterolateral and superolateral contact mechanics of acetabulum and lead to more risk of cam-type FAI. As for the concavity of head-neck junction, because OA subjects had bigger alpha, beta, gamma and delta angle, the average concavity of OA subjects was less than that of the normal subjects which means more aspherical femoral heads and more risk of cam-type FAI. It demonstrated that cam-type FAI resulted in substantially elevated contact pressures and von Mises stresses at the acetabular cartilage^28^. Moreover, cam-type FAI does not only directly damage the acetabular cartilage and labrum but also affects the hip joint mechanical loading. Peak maximum shear stresses were found at the anterosuperior region of the underlying bone during squatting in acetabulum with cam-type FAI, and the peaks at the anterosuperior acetabulum were substantially higher for the patients with cam FAI (15.2 ± 1.8 MPa) in comparison with the controls (4.5 ± 0.1 MPa). These peaks were not situated on the cartilage, but located on the underlying bone^29^. Thus, these may be the reasons for a higher prevalence of hip OA in OA group.

Because OA subjects had smaller acetabular and neck anteversion angles in comparison with the normal subjects, OA subjects had a more retroverted tendency of acetabulum and femoral neck. That would lead to superomedial contact pattern between femoral head and acetabulum, and altering mechanics distribution within hip joint^30^. In addition, these characteristics would also increase the risk of pincer-type FAI in the motion of internal rotation and flexion of the hip joint. Besides, OA subjects had larger acetabular inclination and CE angles, these would increase the risk of pincer-type FAI also, and altering the contact mechanics within hip joint, just as the orientation of acetabular sourcil can be a significant predictor of OA location (craniolateral sourcils were more likely to develop lateral rather than medial OA and craniomedial sourcils were more likely to develop medial OA)^31^. In this study, acetabular dysplasia samples were excluded. However, acetabular dysplasia with insufficient acetabular coverage might lead to instability and abnormal mechanics patterns within the hip joint which would cause hip OA too^32^. Therefore, acetabular overcoverage and insufficient coverage were both risk factors for hip OA. Optimum femoral head coverage by the acetabulum of hip joint was required for long lasting pain free functioning. In our opinion, taking CE angle as an example, there might be a safe range, below/above a specific minimum/maximun threshold would increase the risk of hip OA. But the precise range needs to be further explored. Further comparison of the contact mechanics between normal and OA samples may provide insight into the pathogenisis of hip OA.

As for gender differences, males had, on average, greater AP and lateral physeal angles, that indicated less abduction and anteversion of femoral heads than that of the females. Regarding concavity, males had, on average, larger alpha, gamma and delta angle which mean less concavity at the anterior, superior and inferior head-neck junction (less sphericity of the femoral head) than that of the females. In addition, on average, males had greater femoral head coverage in comparison with the females. Greater coverage and less sphericity of the femoral heads might account for the higher prevalence of OA in Chinese males than that of the females^19^.

Besides, the incidence of OA has racial and geographic distribution differences^33^. Nevitt and Zhang^19^,^34^ reported that the prevalence of hip OA among Chinese subjects in Beijing was lower than that of the Caucasians in the United States. In addition, the rate of total hip replacement was much higher in Caucasians than Asians in a mixed ethnic population from Hawaii^35^. When compared to the study by Toogood^21^, the ratios of AOS/POS and SOS/IOS were greater in Chinese than that of the Caucasian subjects, indicating femoral heads of the Chinese subjects tended to be translated anteriorly and superiorly which were beneficial to avoid FAI. These were different from the Caucasians, which tended to be translated anteriorly and inferiorly. In addition, the alpha, beta, gamma and delta angle were all smaller in Chinese subjects, which mean Chinese had larger head-neck concavity at the anterior, posterior, superior and inferior head-neck junctions (a more spherical femoral head). Besides, the average neck anteversion angle was greater in Chinese subjects. As reported, greater head-neck offset, head-neck concavity, and anteversion of the femoral neck^18^,^22^ could help to avoid cam-type FAI.

Furthermore, less acetabular coverage of femoral heads were found in Chinese subjects. The average acetabular inclination angle of Chinese subjects (male: 36.21°, female: 34.36°) was smaller than that of the Caucasians reported by Nakahara (male: 36.4°, female: 39.1°)^25^. The mean CE angle was smaller in Chinese subjects (male: 31.67°vs 37.9°; female: 28.91°vs 34.7°) also^25^. Thus, the acetabulums were shallower in Chinese subjects compared with the Caucasians. This would provide greater range of motion of the hip joint, and lower probability of pincer-type impingement. Decreased acetabular anteversion could be a reason for hip OA too^18^. In this study, we found greater acetabular anteversion angle in Chinese subjects compared with Caucasian subjects (male: 20.10° vs 17.5°, female: 34.36° vs 21.3°)^25^. These characteristics of anatomical morphology may result in a lower probability of pincer-type FAI in Chinese subjects. Except for FAI, these anatomical differences may also result in different contact mechanics within the hip joint which still need to be further explored.

In conclusion, anatomical factors may play an important role in the pathogenesis of hip OA. The anatomical morphology of the hip joint, such as femoral head-neck relationship, sphericity of the femoral head, acetabular coverage, acetabular and femoral neck anteversion may affect the pathogenisis of hip OA. These anatomical factors may be the reasons for racial, geographic distribution and gender differences also.

## Additional Information

**How to cite this article**: Zeng, W.N. *et al*. Investigation of association between hip morphology and prevalence of osteoarthritis. *Sci. Rep.* **6**, 23477; doi: 10.1038/srep23477 (2016).

## Figures and Tables

**Table 1:** Demographics of the study population.

Sample	Gender	Age (yrs)		Weight (kg)	Height (cm)	BMI
		Mean ± SD	Range			
Normal	Male (n = 49)	38.4 ± 8.2	20–50	64.9 ± 8.3	167.7 ± 6.2	23.1 ± 2.8
	Female (n = 44)	37.7 ± 6.7	25–49	54.2 ± 9.1	156.7 ± 5.4	22.0 ± 3.3
OA	Male (n = 36)	37.1 ± 8.4	21–48	67.3 ± 7.0	170.5 ± 4.3	23.2 ± 2.6
	Female (n = 30)	38.0 ± 7.1	24–49	58.0 ± 8.4	159.3 ± 4.1	22.9 ± 3.5

(Mean ± SD).

**Table 2:** Summary of measurements for the head-neck translation and rotation.

		Normal	OA	*p* value	
AOS/POS	Male	1.18 ± 0.23	1.10 ± 0.16	*0.011*	
	Female	1.26 ± 0.26	1.11 ± 0.21	<*0.001*	
* p* value		*0.025*	*0.759*		
SOS/IOS	Male	1.08 ± 0.14	0.93 ± 0.14	<*0.001*	
	Female	1.12 ± 0.17	0.99 ± 0.12	<*0.001*	
* p* value		*0.027*	*0.009*		
AP physeal angle (°)	Male	79.16 ± 7.18	84.11 ± 7.60	<*0.001*	
	Female	77.10 ± 5.90	82.35 ± 8.04	<*0.001*	
* p* value		*0.035*	*0.201*		
Lateral physeal angle (°)	Male	86.07 ± 6.17	88.13 ± 6.77	*0.040*	
	Female	85.03 ± 5.92	87.38 ± 8.25	*0.060*	
* p* value		*0.240*	*0.570*		

**Table 3:** Summary of measurements for the head-neck junction concavity.

		Normal	OA	*p* value
Alpha angle (°)	Male	39.61 ± 2.56	41.42 ± 2.51	<*0.001*
	Female	38.77 ± 2.27	40.46 ± 1.52	<*0.001*
* p* value		*0.020*	*0.008*	
Beta angle (°)	Male	39.51 ± 2.55	40.38 ± 1.90	*0.016*
	Female	39.01 ± 1.88	40.13 ± 1.84	<*0.001*
* p* value		*0.125*	*0.455*	
Gamma angle (°)	Male	47.82 ± 2.19	48.96 ± 2.28	*0.001*
	Female	47.05 ± 2.94	48.09 ± 2.69	*0.032*
* p* value		*0.047*	*0.046*	
Delta angle (°)	Male	38.91 ± 1.93	40.15 ± 2.25	<*0.001*
	Female	38.29 ± 2.13	39.30 ± 2.22	*0.006*
* p* value		*0.040*	*0.031*	

**Table 4:** Summary of measurements for the acetabula and femoral neck anteversion.

		Normal	OA	*p* value
Acetabular anteversion angle (°)	Male	20.10 ± 4.62	15.93 ± 4.64	<*0.001*
	Female	23.19 ± 8.40	16.54 ± 3.94	<*0.001*
* p* value		*0.003*	*0.429*	
Acetabular inclination angle (°)	Male	36.21 ± 3.62	38.22 ± 3.62	<*0.001*
	Female	34.36 ± 3.62	37.09 ± 3.69	<*0.001*
* p* value		*0.001*	*0.080*	
CE angle (°)	Male	31.67 ± 6.42	33.53 ± 5.08	*0.036*
	Female	28.91 ± 6.73	31.13 ± 5.63	*0.037*
* p* value		*0.005*	*0.011*	
Neck anteversion angle (°)	Male	16.81 ± 7.09	15.02 ± 7.53	*0.114*
	Female	20.57 ± 6.40	18.07 ± 6.82	*0.024*
* p* value		<*0.001*	*0.017*	

**Table 5:** Intra- and inter-observer reproducibility of each anatomical parameter.

	Intra-Observer		Inter-Observer	
	Mean difference	95% LOA	Mean Difference	95% LOA
AOS/POS	−0.01	−0.17 to 0.15	−0.01	−0.16 to 0.13
SOS/IOS	−0.09	−0.21 to 0.03	−0.05	−0.14 to 0.04
AP physeal angle (°)	−0.64	−4.47 to 3.19	−0.11	−2.74 to 2.52
Lateral physeal angle (°)	−0.70	−4.18 to 2.78	−0.23	−2.62 to 2.15
Alpha angle (°)	−0.28	−3.33 to 2.77	−0.27	−2.66 to 2.12
Beta angle (°)	0.15	−3.12 to 3.42	0.14	−1.64 to 1.92
Gamma angle (°)	−0.24	−3.23 to 2.74	0.16	−2.28 to 2.61
Delta angle (°)	−0.09	−3.18 to 3.00	0.15	−2.62 to 2.92
Acetabular anteversion angle (°)	−0.15	−2.17 to 1.88	−0.21	−1.50 to 1.07
Acetabular inclination angle (°)	−0.42	−3.27 to 2.43	−0.09	−2.46 to 2.28
CE angle (°)	−0.17	−3.20 to 2.86	0.02	−2.48 to 2.52
Neck anteversion angle (°)	−0.29	−2.82 to 2.24	−0.07	−1.61 to 1.48
